# Examining Predictors of Myocardial Infarction

**DOI:** 10.3390/ijerph182111284

**Published:** 2021-10-27

**Authors:** Diane Dolezel, Alexander McLeod, Larry Fulton

**Affiliations:** 1 Health Information Management Department, Texas State University, San Marcos, TX 78666, USA; dd30@txstate.edu; 2 Computer Information Systems & Quantitative Methods Department, Texas State University, San Marcos, TX 78666, USA; am@txstate.edu; 3 School of Health Administration, Texas State University, San Marcos, TX 78666, USA

**Keywords:** myocardial infarction, prediction, cardiovascular

## Abstract

Cardiovascular diseases are the leading cause of death in the United States. This study analyzed predictors of myocardial infarction (MI) for those aged 35 and older based on demographic, socioeconomic, geographic, behavioral, and risk factors, as well as access to healthcare variables using the Center for Disease (CDC) Control Behavioral Risk Factor Surveillance System (BRFSS) survey for the year 2019. Multiple quasibinomial models were generated on an 80% training set hierarchically and then used to forecast the 20% test set. The final training model proved somewhat capable of prediction with a weighted F1-Score = 0.898. A complete model based on statistically significant variables using the entirety of the dataset was compared to the same model built on the training set. Models demonstrated coefficient stability. Similar to previous studies, age, gender, marital status, veteran status, income, home ownership, employment status, and education level were important demographic and socioeconomic predictors. The only geographic variable that remained in the model was associated with the West North Central Census Division (in-creased risk). Statistically important behavioral and risk factors as well as comorbidities included health status, smoking, alcohol consumption frequency, cholesterol, blood pressure, diabetes, stroke, chronic obstructive pulmonary disorder (COPD), kidney disease, and arthritis. Three access to healthcare variables proved statistically significant: lack of a primary care provider (Odds Ratio, OR = 0.853, *p* < 0.001), cost considerations prevented some care (OR = 1.232, *p* < 0.001), and lack of an annual checkup (OR = 0.807, *p* < 0.001). The directionality of these odds ratios is congruent with a marginal effects model and implies that those without MI are more likely not to have a primary provider or annual checkup, but those with MI are more likely to have missed care due to the cost of that care. Cost of healthcare for MI patients is associated with not receiving care after accounting for all other variables.

## 1. Introduction

Cardiovascular diseases (CVDs) continue to be the leading causes of death in the United States [1]. Four out of five deaths from CVDs are due to myocardial infarctions (MI) or strokes [2]. An acute myocardial infarction, also called a heart attack, occurs when blood flow to the heart is obstructed causing damage to the heart muscle [3]. The number one cause of an MI is Coronary Artery Disease (CAD) which occurs when plaque, often created by cholesterol deposits, buildup on the coronary arteries supplying the heart with blood [4]. The plaques block the blood flow to the heart causing an artery to narrow and harden (atherosclerosis), leading to a heart attack. Other equivalent terms for CAD are coronary heart disease or ischemic heart disease [5].

In 2017, Myocardial infarctions accounted for 110,346 deaths in the United States [3,6]. Approximately 25% of all MI victims die before reaching a hospital [7]. Serious complications can occur among MI survivors that may include heart failure, cardiogenic shock, or death [7]. The prevalence of myocardial infarction is closely monitored and the Centers for Medicare and Medicaid Services (CMS) hospital value-based purchasing programs have required reporting of Acute Myocardial Infarction (AMI) 30-Day Mortality Rates since 2011 [8].

### 1.1. Risk Factors

Prior research indicates that demographic factors such as race, gender, ethnicity, and veteran status are associated with the incidence of myocardial infarctions [9,10,11,12]. Males generally have higher risk for MI [13] along with African American/Blacks [14], unmarried individuals [15,16], and veterans [17].

Socio-economic risk factors such as income, education, marital status, and employment are associated with incidence rates of MI. Low income status [18], lower education levels [19], and lack of home ownership (a proxy for wealth) [20] have all been associated with higher risk of MI. Unemployment is also associated with higher risk [21].

Geography may be an important factor in estimating incidence rates for MI [22]. One study suggested that counties with low income are associated with higher incidence rates [23]. Another study suggested that increased geographical disparities may exist [24]. Rural patients are more likely to die from an acute MI (AMI) [25].

Behavioral and risk factors (comorbidities) for an MI are known to include high blood pressure, high cholesterol, diabetes, obesity, exercise, tobacco use, age, general health, mental health, and depression [6,26]. Self-reported poor health status [27], smoking status [28], high cholesterol [29], high blood pressure [30], diabetes [31], days physical health poor not good [27], stroke [32], chronic obstructive pulmonary disorder-COPD [33], kidney disease [34], and arthritis [35,36] have all been identified as risk factors for MI. Drinking alcohol in moderation has also been shown to reduce the risk of MI; however, excess drinking increases the risk [37]. Psychological factors related to having an MI include mental health and depressive disorders [38].

Healthcare access factors may be associated with MI incidence. Financial concerns such as not having healthcare coverage sometimes delay presentation for MI [39] and may perhaps even prevent diagnoses. Having no primary healthcare provider(s) or annual checkup have also been associated with comorbidities such as diabetes [40].

### 1.2. Purpose and Significance

The purpose of this research was to examine and model the factors contributing to myocardial infarction to help guide individuals toward early preventative behaviors. The significance of this study is that it provides a comprehensive look at MI occurrence with multiple models based on existing literature and new data coupled with specific hypotheses regarding access. Because of the high incident rate of occurrence of MIs and the potential for serious complications, reviewing known risk factors and identifying new ones is important and contributes to the reduction of mortality rates.

## 2. Materials and Methods

This research developed explanatory and predictive estimates for the prevalence of myocardial infarction for the population 35 and older using the 2019 Behavioral Risk Factor Surveillance System-BRFSS [41] survey based on demographic, socioeconomic, geographical, risk behavior, and access variables. Based on previous studies, we expected that demographics including male status [13], African American/Black status [14], unmarried status [15,16], and veteran status [17] would be associated with MI risks. Based on previous research, low income status [18], education [19], and home ownership (a proxy for wealth) [20] were also included as socioeconomic factors. Geographical variation was shown to be somewhat relevant in the prediction of MI [21,22] and thus was included in our analysis. Further, risk factors such as self-reported poor health status [27], smoking status [28], regular alcohol consumption with potential quadratic effects [13,37], high cholesterol [29], high blood pressure [30], diabetes [31], days physical health poor not good [27], stroke [32], chronic obstructive pulmonary disorder-COPD [33], kidney disease [34], and arthritis [35,36] were also included. Finally, we anticipated that access to care due to cost [39] or other access factors representing potentially undiagnosed MI’s (no personal provider(s) or annual checkup) would help explain and predict MI status.

### 2.1. Data and Software

The 2019 BRFSS data provided a cross-sectional view of the United States population. This instrument, which is moderately reliable, collects information regarding health behaviors from participants [42]. When weights are applied, the BRFSS data provides population estimates [43]. There are three weights that BRFSS uses: individual, strata, and sampling unit. The individual weight balances (rakes) for eight separate groups: age group by gender, race/ethnicity, education, marital status, tenure, gender by race/ethnicity, age group by race/ethnicity, and phone ownership. Strata weights are calculated using the number of available records versus the number of total records within each geographic and density stratum. Cluster weights are based on estimates of the proportion of population sampled from each primary sampling unit (e.g., cluster of telephone numbers). The general formula for the BRFSS weighting scheme is Equation (1) [43].

$$Final\;Weight=\frac{S\;\times A\times P}{N}\;$$

In Equation (1), the *S* is the stratum weight, *A* is the number of adults in the in the household, *N* is the number of residential landlines in the household, and *P* is the post-stratification raking for the groups mentioned previously.

Both Anaconda Python [44] and R Statistical Software version 4.03 [45] were used for all computations. The R survey package [46] provided the complex weighting and quasi-binomial (QB) analysis. Other libraries and packages used in the analysis are available online via Github.com and RPubs.com [47,48].

### 2.2. Sample Size

For this study, we focused on the population most at risk, those aged 35 and older, thus the BRFSS data were filtered accordingly. After filtering, there were 349,261 observations. We then screened the data for missing by row (observation) and column (variable). Eighty-five observations (rows) were eliminated due to greater than 20% missing responses, leaving 349,176 total observations. When weighted, these 349,176 represented a population of 177,573,632 aged 35 and older. No variables (columns) were excluded, as the maximum missing from any column was less than 4% with the total missing values equal to 0.48%. Because of the small number of missing items, measures of center (medians/modes) were imputed for the remaining missing data.

### 2.3. Dependent Variables

The dependent variable of interest in the BRFSS dataset was ‘CVDINFR4,’ Has a doctor, nurse, or other health professional ever told you that you had a heart attack, also called a myocardial infarction?’ The coding for this variable was 0 = No, 1 = Yes, 7 = Don’t Know, and 9 = Refused. About 0.7% of the respondents indicated that they did not know or refused to answer. These respondents were re-coded into the modal response, ‘No’, which then accounted for 91.1% of the observations, thus producing an imbalanced, dichotomous variable with 0 = No or unknown and 1 = Yes.

### 2.4. Independent Variables

#### 2.4.1. Demographic Variables

Age. The ‘_AGE_G’ variable in the BRFSS dataset provides six-level imputed age categories (18–24, 25–34, 35–44, 45–54, 55–64, and 65+). Categories 3, 4, 5, and 6 (age groups 35 and older) were retained for this analysis, as these groups accounted for 98.5% of all MI observations. The referent class was the youngest age group, ages 35–44.

Race. The ‘IMPRACE’ variable from the CDC dataset was used for analyzing race (Imputed race/ethnicity value). The factor levels for this variable were White Non-Hispanic, Black Non-Hispanic, Asian, Non-Hispanic, American Indian/Alaskan Native Non-Hispanic, Hispanic, and Other Race Non-Hispanic. From this measure, we produced dichotomous variables indicating ‘Black,’ ‘Hispanic,’ and ‘Other’ were built. The reference class was ‘White.’ We expected that minorities would have higher risk of MI incidence similar to some previous studies [49].

Gender. The BRFSS data include a variable representing gender (‘SEXVAR’, sex of respondent). Females served as the referent category, as males incident rates for MI are generally higher [50].

Marital Status. A common finding of studies is that marital status is associated with incidence rates of MI. Unmarried status is associated with higher incidence rates [16]. Using the ‘MARITAL’ variable in the BRFSS data set (with levels married, divorced, widowed, separated, never married, a member of an unmarried coupled, and refused), we coded three dichotomous variables: previously married (divorced, widowed, separated), never married, and married/member of an unmarried couple. The referent group was married/member of an unmarried couple as we expected odds ratios greater than 1.0 for the other categories. About 0.84% refused to answer this question, and these observations were placed with the referent group, which was the modal response.

Veteran Status. Previous research has documented that veterans are at higher risk for multiple disorders including MI [17,51]. Thus, we included veteran status (the BRFSS ‘VETERAN3’ variable). This variable was defined as active duty service in the United States Armed Forces either in a regular military or National Guard or military reserve unit and was coded as 1 = Yes, 2 = No, 7 = Don’t Know/Not Sure, 9 = Refused. We recoded this variable as 1 = known veteran and 0=otherwise (referent group and modal response). About 0.25% did not know or refused, and these observations were added to the referent group, not a veteran.

#### 2.4.2. Socioeconomic Status Variables

Income. Income was derived from the ‘INCOME2’ BRFSS categorical variable and collapsed to four levels: do not know or refused to answer, less than $25,000 US dollars, less than $75,000 US dollars, and $75,000 or more (referent and modal category). Do not know and refusals were modeled and not placed with the modal response, as they represented 11.4% of the observations.

Education. Highest level of education attained, calculated from the ‘EDUCA’ BRFSS variable, included four levels: pre-high school education, high school education, post-high school but incomplete college education, and college education (referent and modal category). Those who refused to answer (0.06%) were placed with the modal response (college educated).

Employment Status. Work status, obtained from the BRFSS ‘EMPLOY1’ variable, was measured as employed for wages or self-employed (referent and modal category), retired or unable to work, out of work for any length of time, and not working for other reasons. Refusals to answer (0.08%) were placed with the referent and modal group.

Rent Home. Home ownership derived from the ‘RENTHOM1’ BRFSS variable (do you own or rent your home) was coded dichotomously, with 1 = Own Home (referent and modal group) and 2 = Otherwise. About 0.08% were missing and placed with the referent group.

#### 2.4.3. Geographical Variables

Census Divisions. State-level data (‘STATE’ BRFSS variable) were aggregated at the Census Division level for use in modeling (9 divisions), while territories were analyzed together as a 10th division (referent group). The divisions included New England, Middle Atlantic, East North Central, West North Central, South Atlantic, East South Central, West South Central, Mountain, Pacific, and territories. Table 1 provides the states by census division.

##### Metropolitan Status

Metropolitan status (‘_METSTAT’ from the BRFSS) was a dichotomous variable for metropolitan status of the county coded with 1 indicating a metropolitan area (modal group) and 0 indicating otherwise (referent group).

#### 2.4.4. Behavioral and Health Status Variables

##### Comorbidities 

The following variables (and their associated BRFSS variables) were measured dichotomously with 1 = comorbidity and 0 = otherwise (referent and modal response for all variables): Asthma (‘ASTHMA3’), stroke (‘CVDSTRK3’), depression (‘ADDEPEV3’), pre-diabetic status (‘DIABETE4’), diabetic status (‘DIABETE4’), high BMI (‘_RFBMI5’), high blood pressure (‘_RFHYPE5’), high cholesterol (‘TOLDHI2’), skin cancer (‘CHCSNCR’), cancer (‘CHCOCNR’), COPD (‘CHCOPD2’), kidney disease (‘CHCKDNY2’), and arthritis (‘HAVARTH4’). In all cases, do not know and refused responses were coded as the referent category.

##### Behavioral Variables 

Dichotomous behavioral variables included poor exercise (‘__PA300R3’, less than 301 or more minutes of exercise weekly), smoking 100 cigarettes or more in lifetime (‘SMOKE100’), and chewing or using snuff (‘USENOW3’). Quantitative behavioral variables, all min–max scaled between 0 and 1, included the number of days mental health was poor during the month (recode of ‘MENTHLTH’), the number of days physical health was poor during the month (recode of ‘PHYSHLTH’), the days alcohol was consumed (recode of ‘ALCDAY5’), and this same percentage squared. Min–max scaling produces a proportion for mental health, physical health, and days alcohol was consumed. The reason for including a quadratic component for the percent of days alcohol was consumed is that previous studies have shown there is a cardio benefit to some drinking, but a deleterious effect when that drinking is excessive [37].

##### Health Status

Health status (‘GENHLTH’) was measured as poor health, fair health, good health, or better than good health (modal and referent category). Do not know and refused responses (0.3%) were coded as better than good health.

#### 2.4.5. Healthcare Access Variables

##### No Health Plan 

The BRFSS variables ‘HLTHPLN1’ (Do you have any kind of health care coverage, including health insurance, prepaid plans such as HMOs, or government plans such as Medicare, or Indian Health Service?) was used as one of the measures of healthcare access. This dichotomously coded variable (0 = Health Plan, referent, and modal response, and 1 = No Health Plan) included 0.4% do not know and refused responses coded as the referent and modal response.

##### Personal Doctor(s) 

‘PERSDOC2’ from BRFSS (do you have one person you think of as your personal doctor or health care provider) was initially coded as 1 = Yes, only one, 2 = More than one, 3 = No, 7 = Don’t Know, 9 = Refused. We recoded this variable dichotomously with 0 = at least one doctor (modal and referent), 1 = no doctor. Missing (0.04%) were included with the modal/referent group.

##### Checkup Status 

‘CHECKUP1’, about how long has it been since you last visited a doctor for a routine checkup (1 = within past year, 2 = within past 2 years, 3 = within past 5 years, 4 = five or more years, 7 = Do not know/Not sure, 8 = Never, 9 = Refused) was recoded into a dichotomous variable where 1 = known checkup within last year and 0 = no checkup within the last year or unknown status.

##### Cost Prevented Care 

The BRFSS variable, ‘MEDCOST’, measured if there was a time in the past 12 months when the respondent needed to see a doctor but could not because of cost with 1 = Yes and 0 = Otherwise (referent and modal category). Do not know and refused responses (0.08%) were coded as the modal response. We calculated the proportions for each group variable of the unweighted versus weighted values and provide them in Table 2.

### 2.5. Inferential Statistics

#### 2.5.1. Statistical Modeling 

The dependent variable of interest, ‘MI’, was coded dichotomously. With complex sampling weights applied, the binomial response becomes fractional, and thus the quasi-binomial (QB) distribution which allows for non-integer response variables is an appropriate model. In fact, the use of a logistic regression model based on a binomial link is not possible [52]. The QB (Equation (2)) also estimates variance in the data not totally explained by the binomial (Fox & Weisberg, 2018) [53].

$$P\left(X=k\right)=\left(\begin{array}{c} N\\ k \end{array}\right)p{\left(p+k\phi \right)}^{k-1}{\left(1-p-k\phi \right)}^{N-k}$$

In this equation, N is the count of the observations post weighting, p is the dependent variable occurrence probability, k tallies the number of successes, and ϕ is an added variance parameter not present in the binomial. When the value of ϕ is zero, the equation is the standard binomial. The primary advantage of using a quasi-binomial (whether with over-dispersed data or otherwise) is that it allows fractional data (as in the case of weighting) and that if data are not over dispersed it converts directly to the binomial. The assumptions of independence need not hold strictly, as they are estimated by the over-dispersion parameter.

#### 2.5.2. Training and Test Set 

To investigate the model performance, a 20% test set was removed from the data. The QB models were then used to forecast that unadulterated 20% to estimate accuracy, precision (positive predictive value), recall (sensitivity), and the F1 score (harmonic mean of precision and recall) statistics.

#### 2.5.3. Models 

**Figure 1:**
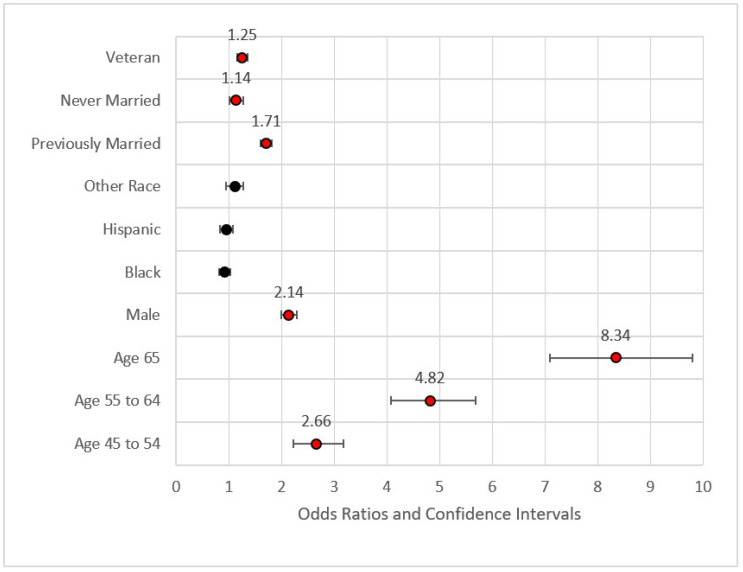
Forest plot of Model 1 (red = significant odds ratios greater than 1.0, black = not significant).

QB models were built based on variable grouping and variable significance as well as overall. Seven models were built on the training data: (1) demographic variables, (2) demographic + socioeconomic variables, (3) demographic + socioeconomic + geographic variables, (4) demographic + socioeconomic + geographic + health status/behavioral risk variables, (5) demographic + socioeconomic + geographic + health status/behavioral risk variables + access variables, (6) complete model with significant variables only, and (7) complete model. After each level, only significant variables at the α = 0.05 level were retained for the next analysis. Model 6, the reduced model with significant variables, was then used to predict the unadulterated, randomly selected test set. The F1-score, the harmonic mean of precision (positive predictive value) and recall (sensitivity), was used to evaluate model explanatory capability. Model 6, the complete model with significant variables, was then compared against a model built on the complete dataset to evaluate coefficient stability. Forest plots were used to illustrate the models shown in Figure 1.

#### 2.5.4. Model 1: Demographics (D) 

Figure 1 provides the forest plot for Model 1. This first model included demographic data only. This model identified higher odds ratios of MI for veterans versus non-veterans (Odds Ratio = OR = 1.25, *p* < 0.001), those previously married versus those who were married or a member of an unmarried couple (OR = 1.71, *p* < 0.001), those never married versus those who were married or a member of an unmarried couple (OR = 1.14, *p* < 0.05), those aged 45–54 versus those aged 35–44 (OR = 2.66, *p* < 0.001), those aged 55–64 versus those aged 35–44 (OR = 4.82, *p* < 0.001), and those aged 65 or older versus the referent group (OR = 8.34, *p* < 0.001). None of the race variables were statistically significant in predicting the incidence of MI, a finding congruent with previous research on incidence of MI [54]. Fatalities from MI and other CHD incidents, however, were found to be much higher in the African American community [13]. The effect size for Model 1 was nominal with pseudo-R^2^ (calculated as 1—model deviance/null deviance) equal to 0.083. The maximum variance inflation factor was 7.158 associated with Age 65+ (related to Age 54–65). The statistically significant variables from Model 1 were included in Model 2.

#### 2.5.5. Model 2: D + Socioeconomics (S) 

**Figure 2:**
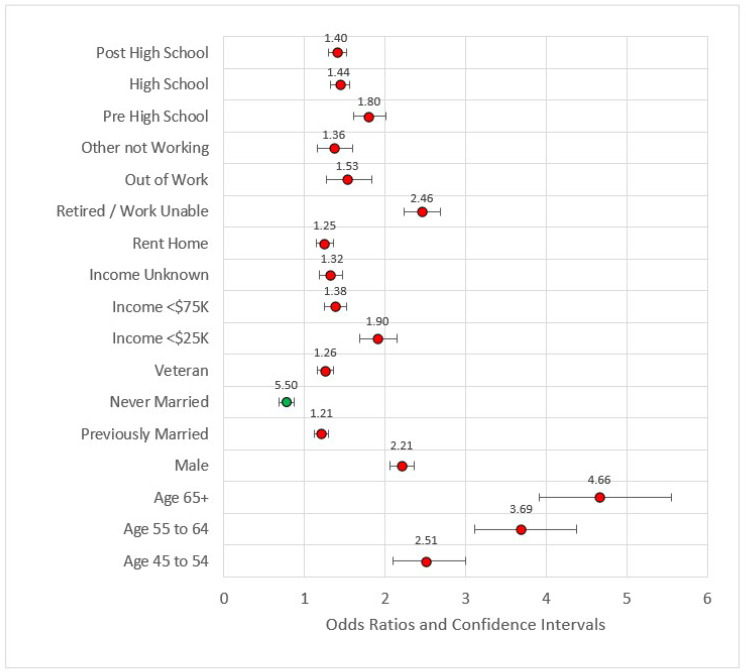
Forest plot of Model 2 (red = significant odds ratios greater than 1.0, green = significant odds ratios less than 1.0, black = not significant).

In Model 2, we added the socioeconomic variables (education, income, employment, and ownership variables). All variables in this model were statistically significant and all were associated with odds ratios greater than one when compared to the referent level except for ‘never married.’ Never married versus married or member of an unmarried couple had OR = 0.77, *p* < 0.001. This is a reversal in the directionality associated with socioeconomic factors such as income and education. Compared to earners making $75,000 U.S. dollars or more, all other categories were at higher risk for MI incidence. Compared to individuals with four or more years of college, all other educational categories were also at higher risk of MI. The effect size of the model was still small (pseudo-R^2^ = 0.117), and the maximum VIF was 8.332. Figure 2 is the forest plot for this model.

#### 2.5.6. Model 3: D + S + Geographical (G) 

**Figure 3:**
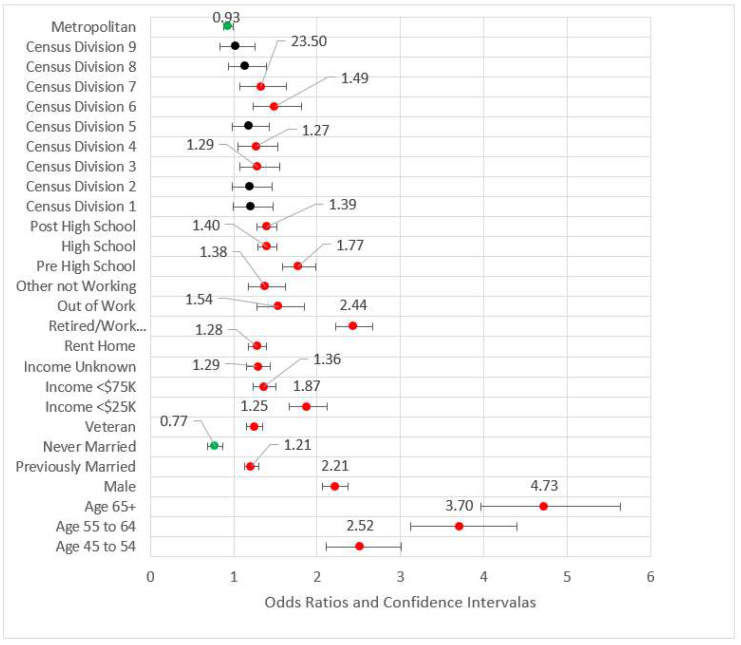
Forest plot of Model 3 (red = significant odds ratios greater than 1.0, green = significant odds ratios less than 1.0, black = not significant).

Model 3 included both previous models’ statistically significant variables and added metropolitan status as well as Census divisions (referent group = Division 10, the territories). All variables retained from Model 2 were directionally consistent with approximately the same magnitudes and statistically significant. For the geographic variables, metropolitan status, and Divisions 3, 4, 6, and 7 (East North Central, West North Central, East South Central, and West South Central, respectively) were statistically significant. For the divisions, all odds ratios were higher than the referent group (Division 10, the territories of Guam and Puerto Rico.) We found no studies that addressed this difference, although Puerto Rico has been shown to have lower CHD incidence than Framingham, Massachusetts as well as Honolulu, Hawaii [55]. While those in metropolitan areas appear to be associated with nominally lower risk than those in non-metropolitan areas (OR = 0.92, *p* < 0.01), the effect in this model is small. Geography added little value to the effect size (pseudo-R^2^ = 0.118), and the maximum VIF was 8.417. Figure 3 is the forest plot of Model 3.

#### 2.5.7. Model 4: D + S + G + Behavioral/Risk Variables (B) 

**Figure 4:**
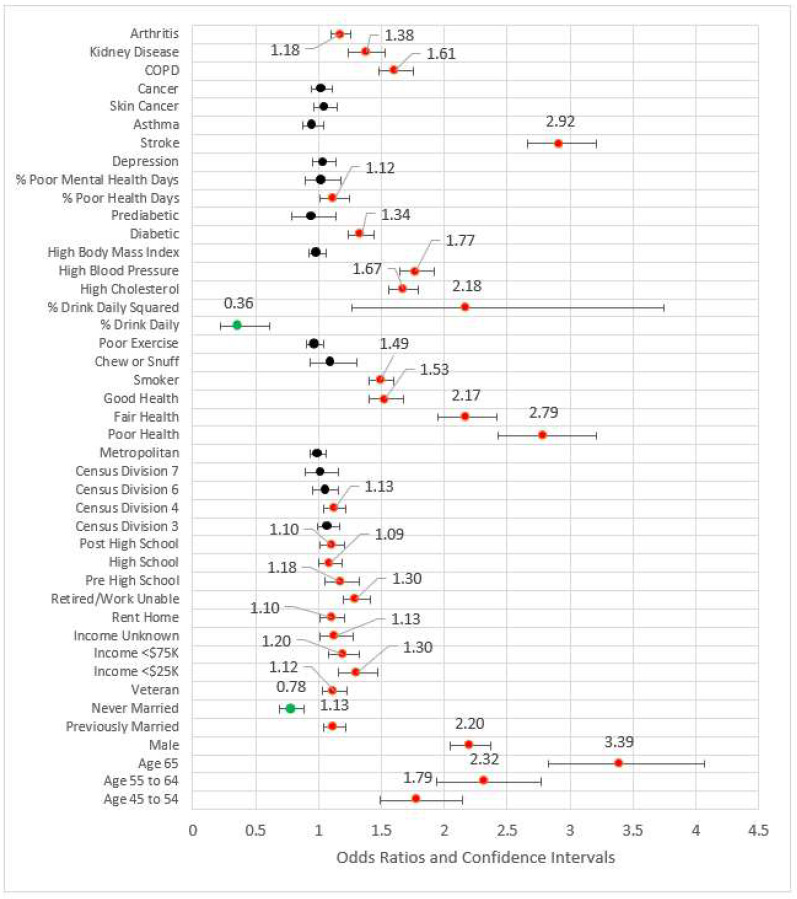
Forest plot of Model 4 (red = significant odds ratios greater than 1.0, green = significant odds ratios less than 1.0, black = not significant).

Model 4 (see Figure 4) added behavioral and risk factors to the previous statistically significant models. Several variables from the previous models fell out. Out of work, other not working, high school, Census Division 3 (East North Central), Census Division 6, Census Division 7, and metropolitan Status were no longer statistically significant at the 0.05 level and thus removed from subsequent modeling. The comorbidities of arthritis, kidney disease, poor physical health days percent, COPD, stroke, diabetes, high blood pressure, and high cholesterol were statistically significant and associated with odds ratios greater than one. Odds ratios for the percent of days an individual consumed alcohol and its squared term were congruent with previous studies, the former coefficient being less than one (OR = 0.37, *p* < 0.001) while the later was greater than one (OR = 2.16, *p* < 0.001). Compared to the referent category of very good or excellent health, all other categories had odds ratios greater than one, indicating higher risk for reportedly worse health. The pseudo-R^2^ for Model 4 was 0.209.

#### 2.5.8. Model 5: D + S + G + B + Access (A)

**Figure 5:**
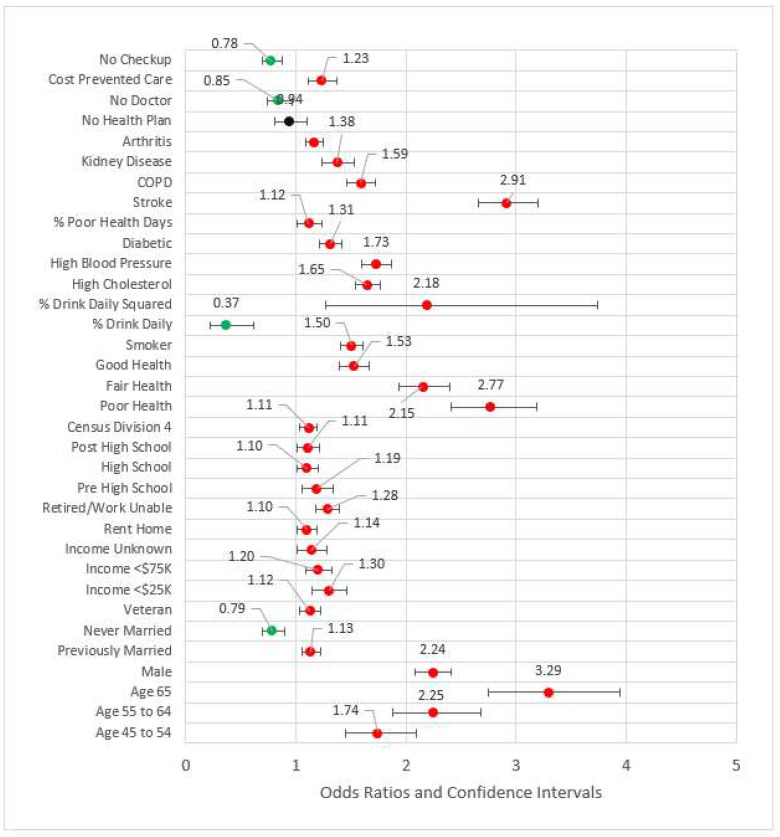
Forest plot of Model 5 (red = significant odds ratios greater than 1.0, green = significant odds ratios less than 1.0, black = not significant).

In Model 5, the four measures of access were added to the significant variables from the previous model blocks (see Figure 5). The odds ratios for not having a primary care physician(s) and not having a checkup in the last year were less than one, possibly indicating those who have had an incidence of MI are followed by physicians. For those who did not obtain healthcare due to cost, the MI odds ratio was 1.23 (*p* < 0.001) versus those who were not financially constrained. Two variables from previous blocks were removed for the subsequent model due to statistical significance: arthritis and out of work status. The effect size, pseudo-R^2^, was 0.210.

#### 2.5.9. Model 6 and Model 7: Statistically Significant Variables and Complete Model 

**Figure 6:**
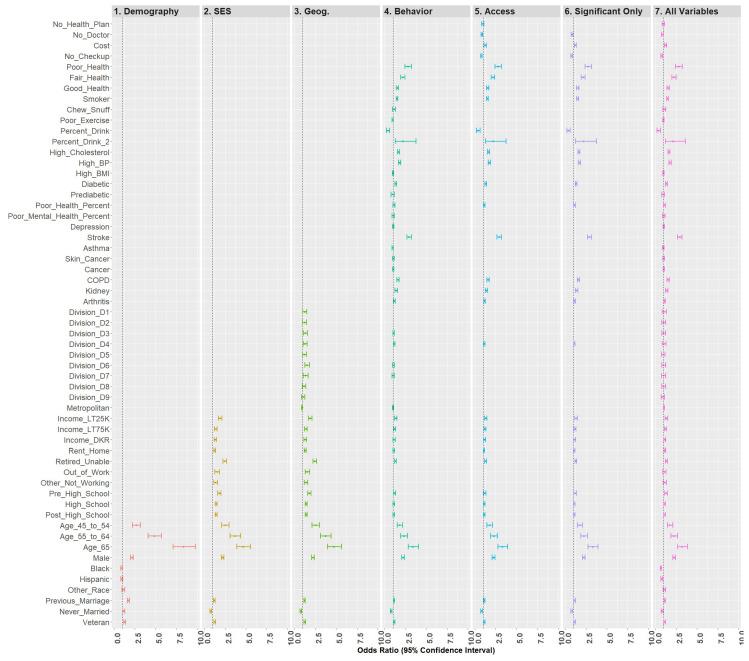
Forest plots of models 1–7.

Model 6 included all statistically significant variables from Models 1 through 5, while Model 7 represented the fully saturated model. The effect sizes for these models (respectively) were 0.210 and 0.212, a nominal difference. Figure 6 is a forest plot depicting all variables from Models 1 through 7. From this figure, it is clear that the coefficients are largely directionally stable and of similar magnitude.

### 2.6. Test Set Predictions

A limitation of weighted sampling with a classification problem is that the unweighted test set sample does not necessarily represent the population. However, comparing model performance or simple analysis performance against different classification metrics provides some information on model utility.

In the case of the QB regression with statistically significant variables only, experimentation with the training set suggested that a classification heuristic would improve model performance on the unseen test set based on the F1 score, the harmonic mean of the precision and recall scores. Specifically, classifying MI status = TRUE when the probability value from the model was 17 or greater produced the highest score. Thus, this heuristic split was used on the test set and generated the metrics in Table 3.

In Table 3, the weighted F1-score is 0.898, which would seem reasonable. However, the F1-score for classifying MI only cases is only 0.339. Under the null model (every case is non-MI), we would classify all non-MIs with 100% accuracy and all MIs with 0% accuracy. Here, the model classifies 92.6% of all non-MIs accurately and 40.6% of all MIs accurately. Despite the low precision (positive predictive values) and recall (sensitivity) statistics for the MI cases, the overall model performs better than the null for the very rare MI observations. Thus, the model has limited utility in classifying positive cases. While we experimented with other classification models including naïve bayes, stochastic gradient descent, random forests, gradient-boosting, extra trees classifiers, discriminant analysis, etc., none were significantly better than the quasibinomial forecasts, and the ability to determine directionality of effect is limited. That analysis is available for review here [48].

### 2.7. Comparison of Training Set and Full Set

**Figure 7:**
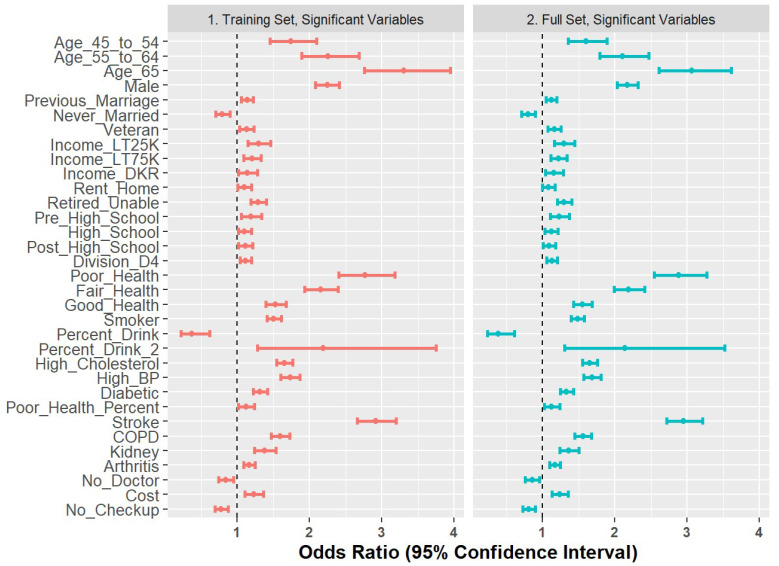
Forest plot of odds ratios for 80% training set versus full set of observations.

As a final step to check coefficient stability, we compared the Model 6 coefficients generated on the training set with those re-built on the full dataset. The results are shown in Figure 7 and demonstrate stability in terms of directionality and magnitude.

## 3. Discussion

Cardiovascular diseases are the leading cause of death in the United States. This research evaluated the demographic, socioeconomic, geographical, behavioral/risk, and access variables. In a large part, the findings were congruent with other research, although there were some novel results.

Demographics were important to all models, with the final model including age, gender, marital status, and veteran status, congruent with previous research [9,10,11,12]. The effect of race fell out as additional variables were added, which supports other research that found lower incidence rates for Blacks and Hispanics, but higher fatality rates [14]. Higher age groups were associated with increased odds ratios for MI, which is an expected finding (control).

Socioeconomic variables remaining in the final model included income, renting rather than owning a home, retirement status, and education. Compared to those making over $75,000 U.S. dollars, those making less money were more likely to have experienced an MI, a finding congruent with previous research [18]. Similar to another study [20], odds ratio estimates for those renting versus those owning their own homes were greater than 1, suggesting increased risk. Those retired or unable to work also had higher odds of MI when compared to those working, supportive of previous research [21]. Lower education status, similar to what has been found previously [18], is also associated with increased risk of MI.

Previous studies found that geography may be an important factor in estimating incidence rates for MI [22] and that rural patients are more likely to die from an acute MI (AMI) [25]. In our study, we found only one Census Division that provided evidence of geographic disparity, Division 4 (West North Central). There were no effects found for metropolitan status in the final model. The reasons for this finding may be due to the inclusion of predictors such as income which reduce the importance of geography.

The remaining risk and behavior variables in the final model included health status, smoking, drinking, high cholesterol, high blood pressure, diabetes, percent of days health was bad, stroke, COPD, kidney disease, and arthritis with odds ratios in the directions to be expected (higher risk for presence of comorbidity or behavior). Unlike previous studies, we found no mental health or depression effects [6,26]. Our findings do support previous research indicating that poor health status [27], smoking status [28], high cholesterol [29], high blood pressure [30], diabetes [31], days physical health poor not good [27], stroke [32], chronic obstructive pulmonary disorder-COPD [33], kidney disease [34], and arthritis [35,36] are risk factors. Interestingly, we found that alcohol consumption exhibited a quadratic effect, supporting previous research [13]. Finally, we found no support for the inclusion of exercising more than 300 min each week or high body mass index, possibly to measurement granularity in the survey.

Most interestingly, we found that three of the four access variables considered in our modeling were important explanatory variables. Financial concerns that resulted in cost preventing care were associated with an increase odds ratio, indicating that MI patients who require care may not receive it. This finding should create some consternation. On the other hand, not having a primary provider or routine checkups had odds ratios lower than one compared to the referent group, indicating that those individuals are less likely to have had an MI. This makes sense when one considers that post-MI (particularly post AMI) checkups and healthcare are necessary. Coupled with the cost finding, the implication is that MI patients are more likely to have primary care providers and checkups but are also more likely to not seek needed care due to cost. This cost finding is congruent with previous research [39].

We also found that models built on the training set were capable of predicting an unseen test set with some success based on classification metrics. Further, we found that the odds ratios were stable for models built on the training set and models built on the full data.

## 4. Conclusions

### 4.1. Significance of Findings

This study is significant for many reasons. First, it evaluates risk factors in a single model to estimate their effects simultaneously. Second, the model uses 2019 data (at the time the most recent data available) to update estimates of odds ratios associated with all variable groupings. Third, the hierarchical development of the models ensures that variance capture from specific and of-interest variable groupings are not over-stated due to failure to include other potentially confounding predictors. Fourth, the model confirms findings associated with some risk factors, but disputes those associated with exercise and mental health/depression effects. Fifth, the mixed findings associated with alcohol use are addressed in that we found a quadratic relationship rather than positive or negative. Finally, we found access differences between respondents reporting an MI and those who did not. In Toto, we believe that this is the most thorough analysis of collective factors associated with MI in the literature.

### 4.2. Limitations

One of the primary limitations of this study is the nature of the data (self-reporting); however, BRFSS is ‘the nation’s premier system of health-related telephone surveys that collect state data about U.S. residents regarding their health-related risk behaviors, chronic health conditions, and use of preventive services [56]. Further, much of the research on MI has involved time-to-event studies [57,58]. With self-reported data, this approach is not feasible, which is a limitation of this study. It is important to note that directionality of odds ratios must be interpreted within the context of each model as well.

### 4.3. Summary

This research confirmed many of the demographic, socioeconomic, behavioral/risk, geographic, and access risk factors associated with the presence of MI, but it also challenged some existing knowledge. For demographics, the incidence rates for minorities were not different from that of the referent group. While this contradicts some research, it is supported by another study that found that fatality rates from MI were higher in minorities, but that incidence rates were not [14]. Further research in this area is required.

The socioeconomic effects present included income, rent versus owning a home, education status, and marital status. While these findings were largely congruent with previous studies, the group that was never married was associated with lower odds ratios for MI. This odds ratio below 1 is consistent even in a marginal model that compares only marital status with MI. The reasons for this finding are unknown and require further research.

Geographic effects were nominal, with only one variable remaining in the final model. Future research should consider the inclusion of geospatial regression analysis at the county level.

Behavioral and risk factor variables were largely congruent with previous studies with some exceptions. Most interestingly, exercising more than 300 min each week and body mass index indicating overweight or obese did not remain in the final model. This may be due to the fact that the granularity of measurement is insufficient, which is a limitation.

Importantly, we found support that access measures are different for those who have experienced an MI versus those who have not. MI patients are more likely to not seek needed care due to cost, but to have primary care providers and health coverage. Taken together, this finding would seem to suggest that while MI patients require additional coverage and checkups, they are sometimes unable to afford the healthcare they require. This cost finding is congruent with previous research [39].

## Figures and Tables

**Table 1:** Census divisions used for estimating geographical impact on MI.

1 = New England	2 = Middle Atlantic	3 = East North Central	4 = West North Central	5 = South Atlantic
Connecticut	New Jersey	Indiana	Iowa	Delaware
Maine	New York	Illinois	Kansas	District of Columbia
Massachusetts	Pennsylvania	Michigan	Minnesota	Florida
New Hampshire		Ohio	Missouri	Georgia
Rhode Island		Wisconsin	Nebraska	Maryland
Vermont			North Dakota	North Carolina
			South Dakota	South Carolina
				Virginia
				West Virginia
**6 = East South** **Central**	**7 = West South** **Central**	**8 = Mountain**	**10 = Territories**	
Alabama	Arkansas	Arizona	Guam	
Kentucky	Louisiana	Colorado	Puerto Rico	
Mississippi	Oklahoma	Idaho		
Tennessee	Texas	New Mexico		
		Montana		
		Utah		
		Nevada		
		Wyoming		

**Table 2:** Proportions by variable group and variable, unweighted (weighted).

Dependent Variable		Behaviors/Risks	
Myocardial Infarction	0.069 (0.057)	Asthma	0.134 (0.133)
**Demographics**		Stroke	0.053 (0.046)
Age 35–44 (Referent)	0.142 (0.231)	Depression	0.183 (0.177)
Age 45–54	0.175 (0.229)	Prediabetic	0.025 (0.026)
Age 55–64	0.241 (0.235)	Diabetic	0.161 (0.150)
Age 65	0.443 (0.305)	High BMI	0.643 (0.646)
Caucasian (Referent)	0.789 (0.662)	High BP	0.463 (0.418)
Black	0.074 (0.114)	High Cholesterol	0.398 (0.370)
Hispanic	0.073 (0.149)	Skin Cancer	0.118 (0.089)
Other Race	0.064 (0.075)	Cancer	0.120 (0.095)
Female (Referent)	0.559 (0.523)	COPD	0.097 (0.084)
Male	0.441 (0.477)	Kidney	0.045 (0.040)
Married (Referent)	0.585 (0.633)	Arthritis	0.388 (0.329)
Previously Married	0.320 (0.260)	Limited Exercise	0.279 (0.274)
Never Married	0.095 (0.107)	Smoker	0.428 (0.420)
Veteran	0.141 (0.120)	Chew or Snuff	0.029 (0.030)
**Socioeconomics**		% Mental Health Days	0.114 (0.121)
<$25 K Income	0.201 (0.203)	% Physical Health Days	0.159 (0.152)
<$75 K Income	0.335 (0.340)	% Days Drinking	0.154 (0.147)
Income-Refused	0.175 (0.165)	% Days Drinking ^2^	0.101 (0.093)
≥$75 K Income-(Referent)	0.290 (0.292)	Poor Health	0.060 (0.060)
Pre-High School	0.073 (0.136)	Fair Health	0.153 (0.159)
High School	0.261 (0.260)	Good Health	0.324 (0.327)
Post High School	0.273 (0.295)	Very Good/Excellent	0.463 (0.454)
College 4+ Years (Referent)	0.394 (0.309)	**Access**	
Working	0.460 (0.537)	No Primary Doctor	0.130 (0.165)
Retired/Unable to Work	0.449 (0.350)	Cost Prevented Care	0.093 (0.116)
Out of Work	0.032 (0.042)	No Annual Checkup	0.169 (0.195)
Other Not Working	0.058 (0.071)	No Health Plan	0.068 (0.102)
Rent Home	0.184 (0.189)		
**Geography**			
Metropolitan	0.683 (0.840)	D6. East South Central	0.063 (0.060)
D1. New England	0.117 (0.049)	D7. West South Central	0.069 (0.118)
D2. Middle Atlantic	0.049 (0.102)	D8. Mountain	0.131 (0.075)
D3. East North Central	0.105 (0.146)	D9. Pacific	0.095 (0.163)
D4. West North Central	0.171 (0.065)	D10. Territories	0.019 (0.011)
D5. South Atlantic	0.182 (0.211)	(*‘D#’* = *division number*)	

**Table 3:** Classification results for Model 7 on the unseen test set.

Metric	No MI	MI	Weighted Average
F1 Score	0.940	0.339	0.898
Precision	0.926	0.406	0.890
Recall	0.954	0.291	0.908
Accuracy	0.926	0.406	0.890
Support	64,976	4860	69,836

## Data Availability

This research used Center for Disease Control Behavioral Risk Factors Surveillance System data available at https://www.cdc.gov/brfss/index.html (accessed on 2 September 2021).
